# Associations of Angiotensin‐Converting Enzyme 2 Activity With Markers of Endothelial Dysfunction and Oxidative Stress in Young Healthy Adults: The African‐PREDICT Study

**DOI:** 10.1161/JAHA.125.047345

**Published:** 2026-10-16

**Authors:** Vuledzani Felicia Maugana, Louise M. Burrell, Sheila K. Patel, Catharina Martha C. Mels, Aletta Elisabeth Schutte, Maserame Cleopatra Mokhaneli, Nonkululeko Hellen Navise, Thabisile Mpofana, Philimon N. Gona, Lebo Francina Gafane‐Matemane

**Affiliations:** ^1^ Hypertension in Africa Research Team (HART) North‐West University Potchefstroom South Africa; ^2^ Department of Medicine Austin Health, University of Melbourne, Heidelberg Melbourne Australia; ^3^ South African Medical Research Council (SAMRC): Extramural Unit for Hypertension and Cardiovascular Disease North‐West University Potchefstroom South Africa; ^4^ School of Population Health University of New South Wales, The George Institute for Global Health Sydney New South Wales Australia; ^5^ Urban Public Health Department, Manning College of Nursing and Health Sciences University of Massachusetts Boston Boston MA USA

**Keywords:** age, angiotensin‐converting enzyme 2 activity, cardiovascular disease risk, endothelial dysfunction, oxidative stress, High Blood Pressure, Hypertension, ACE/Angiotension Receptors/Renin Angiotensin System, Biomarkers, Inflammation

## Abstract

**Background:**

Activation of the renin–angiotensin–aldosterone‐system plays a major role in the pathogenesis of cardiovascular disease through, among others, endothelial dysfunction and oxidative stress. Circulating ACE2 (angiotensin‐converting enzyme 2) is emerging as a biomarker of cardiovascular disease risk; however, its role in younger populations is not well‐established. This study aimed to investigate sex differences in serum ACE2 activity and associations with endothelial dysfunction and oxidative stress‐related markers in young, apparently healthy men and women.

**Methods:**

In this cross‐sectional study, we included 1134 young (aged 20–30 years) men (n=557) and women (n=577). Serum ACE2 activity was measured using a validated fluorometric assay. We analyzed markers of endothelial dysfunction, oxidative stress, and antioxidant capacity. We performed a 2‐sample *t* test and Mann‐Whitney *U* test. We applied multivariable‐adjusted Tobit regression analysis to account for left truncation.

**Results:**

Men had higher ACE2 activity than women (*P*<0.001). After multiple adjustments for covariates, ACE2 activity was positively associated with von Willebrand factor (*P*=0.006), and a disintegrin‐like metalloproteinase with thrombospondin motif type 1 member 13 (*P*=0.019) in men. Among women, ACE2 activity was negatively associated with asymmetric dimethylarginine (*P*=0.043) and superoxide dismutase (*P*=0.004).

**Conclusions:**

Serum ACE2 activity was higher in men than women and showed sex‐specific associations with endothelial dysfunction and oxidative stress‐related markers. Our results suggest an association between ACE2 shedding and pathophysiological processes associated with alterations in endothelial integrity and early endothelial dysfunction, which may be influenced by sex. This highlights the role of circulating ACE2 as a potential early biomarker of cardiovascular disease risk in young adults.

**Registration:**

URL: https://clinicaltrials.gov; Unique identifier: NCT03292094.

**Nonstandard Abbreviations and Acronyms**
African‐PREDICT: African Prospective Study on Early Detection and Identification of Cardiovascular Disease and Hypertension
GPx: glutathione peroxidase
GR: glutathione reductase
ROS: reactive oxygen species
SOD: superoxide dismutase

**Clinical Perspective**

**What Is New?**

- This relatively large study shows the potential relationship between ACE2 (angiotensin‐converting enzyme 2) activity and endothelial function in a young Black and White population, which was not previously established.

**What Are the Clinical Implications?**

- Given the specific role of ACE2 activity in cardiovascular health, our findings suggest that as a biomarker, ACE2 can explain the pathophysiology of cardiovascular disease development through endothelial dysfunction, with varying mechanistic pathways in men and women.

Cardiovascular disease (CVD) is the primary cause of morbidity and mortality globally,^1^ with a higher CVD risk reported in men than women during the premenopausal phase of life.^2^, ^3^ Interactions between genetic and environmental factors may trigger pathways associated with pathophysiological mechanisms such as endothelial dysfunction^4^, ^5^, ^6^ and oxidative stress,^7^ leading to CVD.^8^ Given the early manifestation of endothelial dysfunction that precedes CVD development, assessing circulating biomarkers may provide insights on endothelial functional status and mechanisms underlying the pathogenesis of endothelial dysfunction.^9^, ^10^ Several biomarkers, such as asymmetric dimethylarginine (ADMA), markers of oxidative stress, inflammation, and indices of coagulation are associated with endothelial dysfunction, which is characterized by reduced NO synthesis or bioavailability.^11^, ^12^ Changes in the levels of these biomarkers are known to reflect alterations in the endothelial integrity, thus defined as biomarkers of endothelial function.^11^, ^12^ Therefore, assessment of the markers related to endothelial dysfunction may provide a better understanding of early CVD development and thereby improve cardiovascular risk prediction.

Central to the pathogenesis of CVD, is the heightened activation of the renin–angiotensin–aldosterone system (RAAS),^13^ which mediates endothelial dysfunction and oxidative stress, mainly through the actions of angiotensin II.^14^, ^15^ Angiotensin‐converting enzyme 2 (ACE2), a member of the counter‐regulatory axis of the RAAS, acts to degrade angiotensin II to a bioactive angiotensin heptapeptide (angiotensin [1–7]), thereby limiting the adverse effects of angiotensin II such as sodium and water retention, vasoconstriction, inflammation, oxidative stress, and fibrosis.^16^, ^17^ ACE2 is expressed in several human tissues, including the heart and kidney, and on the surfaces of specific cell types, such as endothelial cells and epithelial cells.^18^, ^19^ Proteolytic shedding of ACE2 from tissues results in a soluble form that can be measured in plasma.^20^, ^21^ Studies have reported that plasma ACE2 activity is undetectable or low in healthy individuals compared with individuals with CVD risk factors.^20^, ^22^ Circulating ACE2 activity is therefore emerging as a biomarker of CVD risk, with several studies showing that elevated plasma ACE2 activity predicts adverse outcomes in individuals with CVD.^23^, ^24^, ^25^

Given that enhanced RAAS activation plays a role in CVD, blockade of the RAAS has been used as a therapeutic target for cardiovascular and renal diseases.^26^, ^27^ Although therapeutic research has primarily focused on blocking traditional components of the RAAS, limited clinical data are currently available on alternative components, such as ACE2, in relation to the pathophysiological processes underlying CVD development. Investigating less‐explored RAAS components like ACE2, alongside markers of endothelial dysfunction and oxidative stress in young, healthy individuals, could enhance early prediction of CVD risk.

Endothelial dysfunction and oxidative stress contribute to the development of CVD, and the observed discrepancies in CVD between men and women can be attributed to sex differences in markers of endothelial dysfunction and oxidative stress. Circulating ACE2 activity was reported to be higher in older men than women with chronic kidney disease,^28^, ^29^ which suggests that this biomarker may be useful in determining how the pathophysiology of CVD differs by sex. However, in young, healthy populations, sex differences in circulating ACE2 activity are not well‐documented, and it is unknown whether ACE2 activity is associated with markers of endothelial dysfunction and oxidative stress, and whether such associations differ based on sex. We therefore aimed to investigate the sex differences in ACE2 activity as well as associations of ACE2 activity with endothelial dysfunction and oxidative stress markers in young, apparently healthy men and women.

## METHODS

### Data Availability Statement

The data that support the findings of this study are available from the corresponding author upon reasonable request.

### Study Design and Participant Recruitment

The African‐PREDICT (African Prospective Study on Early Detection and Identification of Cardiovascular Disease and Hypertension) is a longitudinal study that enrolled a sample of 1202 Black and White young adults (aged 20–30 years) who are being followed up for a period of >10 years to identify novel biomarkers related to early CVD development. In the current study, the term "ethnicity" as opposed to "race" is used to differentiate between the Black and White groups in line with the approved ethics protocol as indicated in the larger study protocol.^30^ Recruitment of participants was conducted in and around Potchefstroom, in North‐West Province, South Africa. Based on the study design, participant recruitment included young, apparently healthy men and women, who self‐reported their ethnicity and race to be African (Black), and those of European (White) descent for comparison. In addition to the balanced selection based on race and sex, participants were also stratified by socioeconomic status (SES). Participants were recruited at workplaces through public advertisements on radio, noticeboards, and newspapers. Volunteers (n=1886) underwent screening to determine eligibility, which required participants to have a clinic brachial blood pressure (BP) of <140/90 mm Hg, to be uninfected with human immunodeficiency virus, have no self‐reported previous diagnosis or were not on medication for any chronic disease, and women to not be pregnant or breastfeeding. Individuals who met eligibility criteria were invited to another appointment for baseline data collection. The median time between screening and data collection was 15 days (interquartile range, 7–40 days). Baseline data were collected between 2013 and 2017 at the Hypertension Research and Training Clinic on the Potchefstroom campus of North‐West University. A total of 1202 (Black: n=606; White: n=596) participants were enrolled in the larger African‐PREDICT study. This substudy used cross‐sectional data for 1134 participants who were also stratified by sex (men: n=557; women: n=577). Participants with missing data for ACE2 activity (n=68) were excluded. The flowchart of the study population in this cross‐sectional study is shown in Figure 1. All participants gave written informed consent before data collection. Ethical approval for the African‐PREDICT study was obtained from the Health Research Ethics Committee of North‐West University (NWU‐00001‐12‐A1), and the study complied with the Declaration of Helsinki criteria for human research. The study is registered at ClinicalTrials.gov (Unique identifier: NCT03292094).

**Figure 1:**
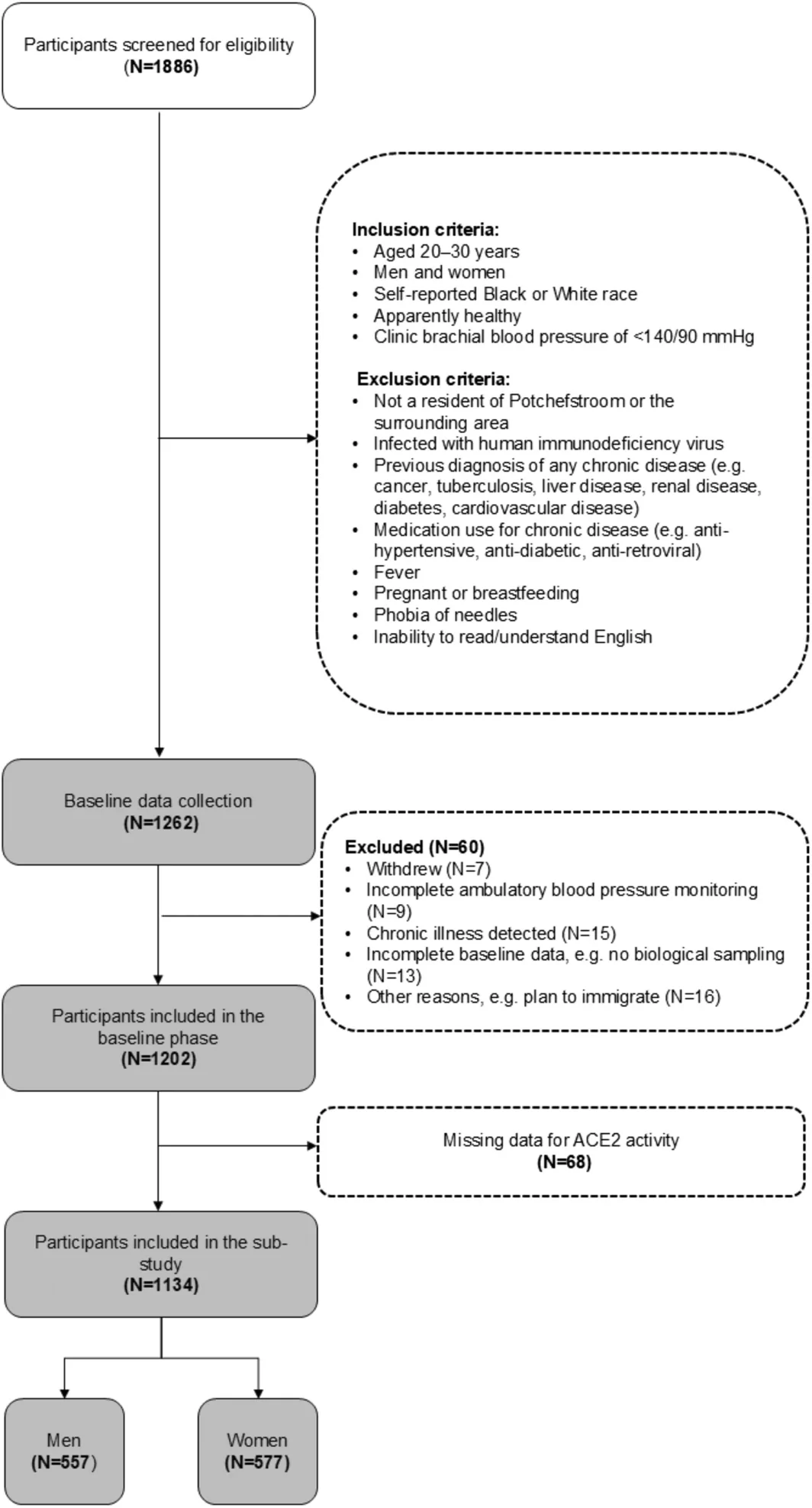
Study population flowchart. ACE2 indicates angiotensin‐converting enzyme 2.

### Questionnaires

A general health questionnaire was used to obtain information on age, sex, ethnicity, smoking status, and alcohol use. Participants' SES was captured. Furthermore, participants were stratified into different SES classes (low, middle, high) using a point‐based system adapted from the Kuppuswamy's Socioeconomic Status Scale to align with the South African context.^31^ Participants were scored in 3 categories: (1) skill level, (2) education, and (3) household income. The South African Standard Classification of Occupation was used to classify skill level. Detailed methods on SES are described elsewhere.^30^, ^32^

### Anthropometric and Physical Activity Measurements

All anthropometric measurements were performed based on the International Standard for Anthropometric Assessment.^33^ Body height (in centimeters) was determined with a SECA 213 stadiometer (SECA, Hamburg, Germany). Participants were requested to remove their shoes and excess clothes (to be dressed in light clothing), and body weight (in kilograms) was then measured with a SECA 813 digital scale (SECA, Hamburg, Germany). Waist circumference (in centimeters) was measured in triplicate with a Lufkin steel anthropometric tape (W606PM, Lufkin, Apex, USA). The median of the 3 measurements was calculated and used in data analysis. Body mass index was calculated in kilograms per square meter. An ActiHeart device (CamNtech, Ltd., Cambridge, United Kingdom) was worn for a maximum of 7 consecutive days, which was used to measure physical activity. Activity energy expenditure was calculated and standardized for body weight.

### Blood Pressure Measurements

A validated BP apparatus (Dinamap Procare 100 Vital Signs Monitor; GE Medical Systems, Milwaukee, USA) with fitted‐sized GE Critikon latex‐free Dura‐Cuffs was used to measure brachial BP and heart rate. After a resting state of 5 minutes, clinic systolic BP, diastolic BP, and heart rate were measured in duplicate on both arms, with 5‐minute intervals between left and right arm measurements, while the participant was in a seated and relaxed state. The mean of the second measurement from the left and right arm was used for further analyses. Participants were requested not to have smoked, exercised, or eaten at least 30 minutes before the BP measurement being taken and were required to be in a seated resting state for 5 minutes with the arm supported at heart level.

Participants were requested to wear an ambulatory device (Card[X]plore device; Meditech, Budapest, Hungary) overnight to measure 24‐hour BP. The ambulatory device was programmed to obtain BP measurements at 30‐minute intervals during the day (6:00 am–10:00 pm) and every hour during the night (10:00 pm–6:00 am). The mean±SD of the successful inflation rate for the study population over a 24‐hour period was 88±12.3%.

### Biological Sampling and Biochemical Analyses

All participants were required to fast overnight for a period of between 8 and 10 hours. Early the next morning, a registered research nurse used a sterilized needle to draw blood from the antebrachial vein, which was stored into different tubes. Whole blood, serum as well as sodium fluoride and EDTA plasma samples were prepared according to standard procedures and stored at −80 °C until the time of analyses.

Basic serum analyses were conducted for total and low‐ and high‐density lipoprotein cholesterol, triglycerides, high‐sensitivity C‐reactive protein, creatinine, and cystatin C (Cobas Integra 400plus; Roche, Basel, Switzerland). Sodium fluoride plasma glucose (Siemens, Erlangen, Germany) was determined (Cobas Integra 400plus). Whole blood EDTA glycated hemoglobin was determined (Cobas Integra 400plus). Cotinine was analyzed using a chemiluminescence method on the Immulite (Siemens, Erlangen, Germany). Estimated glomerular filtration rate was calculated using the Chronic Kidney Disease Epidemiology creatinine and cystatin C formula without the race factor.^34^

Spot urine samples were used to determine albumin (milligrams per liter) and creatinine (millimoles per liter) (Cobas Integra 400plus), and the urinary albumin‐creatinine ratio was calculated.

### Circulating ACE2 Activity

The catalytic activity of ACE2 was measured using a validated, sensitive‐quenched fluorescent substrate‐based assay as previously described.^22^ A volume of 250 μL of serum was diluted into low‐ionic‐strength buffer (20 mmol/L Tris–HCl, pH 6.5) and added to 200 μL ANXSepharose 4 Fast‐Flow resin (Cytiva, Marlborough, MA) to remove an endogenous inhibitor of ACE2 activity.^24^, ^25^ After binding and washing, the resulting eluate was assayed for ACE2 catalytic activity. Duplicate samples were incubated with the ACE2‐specific quenched fluorescent substrate (7‐methoxycoumarin‐4‐acetyl‐Ala‐Pro‐Lys[2,4‐dinitrophenyl]‐OH) (Auspep, Melbourne, VIC, Australia), with or without 100 mM EDTA (Merck, Darmstadt, Germany). Reactions were performed at 37 °C for 200 minutes, continuously monitoring liberated fluorescence using a CLARIOstar Plus plate reader (BMG Labtech, Offenburg, Germany). The rate of substrate cleavage was determined by comparing against a standard curve of the free fluorophore, 7‐methoxycoumarin‐4‐acetic acid (Sigma), and expressed as picomoles of substrate cleaved per milliliter of serum per minute. The lower limit of detection for the ACE2 assay is 0.04 pmol/mL per minute; a total of 283 participants had undetectable ACE2; therefore, they were statistically analyzed as left‐censored for ACE2 to account for the floor effects.^35^, ^36^ The intra‐assay and interassay coefficients of variation were 2.2% and 6.8%, respectively.

### Markers of Endothelial Dysfunction

Urinary ADMA and symmetric dimethylarginine were measured by gas chromatography–mass spectrometry.^37^ Citrate plasma samples were also used to determine von Willebrand factor (vWF) antigen levels (percent antigen) using a sandwich ELISA (DAKO, Glostrup, Denmark). Polyclonal rabbit anti‐vWF antibody and rabbit anti‐vWF‐horseradish peroxidase antibody (DAKO) were used to perform the assay. The Sixth International Standard for vWF/factor VIII was used to determine the standard curve against which the samples were measured. Serum samples were used to measure ADAMTS13 (a disintegrin‐like metalloproteinase with thrombospondin motif type 1 member 13) (nanograms per milliliter) using the MILLIPEX MAP Human Cardiovascular Disease Magnetic Bead Panel 2 (Merck Millipore, Darmstadt, Germany).

### Markers of Oxidative Stress

Total peroxides (one of the measurable reactive oxygen species [ROS]), were determined in serum samples using a high‐throughput spectrophotometric assay and analyzed on a Synergy HT microplate reader (BioTek, Winooski, VT).^38^ For the purposes of this analysis, serum peroxides are referred to as ROS, reported in units, where 1 milligram H_2_O_2_ per liter is equivalent to 1 unit. Serum GGT (γ glutamyl transferase) was analyzed (Cobas Integra 400plus). Spot urine samples were used to measure malondialdehyde by gas chromatography–mass spectrometry and normalized based on creatinine excretion and are expressed as micromolar analyte to millimolar creatinine (micromolar/millimolar). Antioxidant enzymes including superoxide dismutase (SOD) activity (serum), glutathione peroxidase (GPx), and glutathione reductase (GR) (EDTA plasma), as well as total antioxidant status were measured using assay kits (Randox, Antrim, United Kingdom) and the Cobas Integra 400plus (Roche). These assays were performed on a Synergy HT microplate reader (BioTek). Total glutathione was measured using whole blood samples with a Synergy HT microplate reader (BioTek) (Kit: BIOXYTECH GSH/GSSG‐412; Oxis International).

### Statistical Analysis

Statistical analyses were performed using IBM SPSS Statistics, version 31 software (IBM, Armonk, NY) and Statistical Analysis Software, version 9.4 (SAS Institute, Cary, NC). The distribution for all quantitative data was assessed for normality using the Shapiro‐Wilk test, visual inspection of histograms and quintile‐quintile plots. Although several variables violated normality assumptions, a natural‐logarithmic transformation was performed for ACE2 and C‐reactive protein to normalize the distribution. Categorical data (ethnicity, SES class, self‐reported smoking, and alcohol consumption) were presented as frequencies and proportions. Continuous variables were reported as mean±SD if the variables followed a normal distribution and median with 25th to 75th percentiles for variables that were not normally distributed. GraphPad Prism version 8.0.1 was used to construct figures of differences in ACE2 activity, endothelial dysfunction, and oxidative stress‐related markers between men and women. Since ACE2 activity has not been widely studied as an early biomarker of CVD risk and whether sex plays a role in young adults, we conducted sex‐specific analyses using a 2‐sample *t* test, Mann‐Whitney *U* test, and χ^2^ test to determine if there were differences in markers of endothelial dysfunction, oxidative stress, and antioxidant capacity. We applied multivariable‐adjusted Tobit regression analysis to account for left truncation to investigate the association of ACE2 activity with endothelial dysfunction and oxidative stress‐related markers. The Tobit regression procedure was implemented using the Life Regression Procedure in SAS version 9.4. Covariates included age, ethnicity, SES class, BMI, 24‐hour systolic blood pressure, glucose, low‐density lipoprotein, smoking status, alcohol use, and hormonal contraceptive use (women). We used backward elimination to select a parsimonious model. For consistency, the model was applied for all subgroups. Individual multivariable‐adjusted Tobit regression models were run to assess interactions of sex with vWF, ADAMTS13, ADMA, symmetric dimethylarginine, ROS, malondialdehyde, GGT, total antioxidant status, SOD, GPx, GR, and total glutathione. The number of participants with missing values for the main independent variables are indicated in Table S1. Based on the established role of ethnicity in RAAS components^39^, ^40^ and the contribution of hormonal contraceptive use to oxidative stress,^41^, ^42^ statistical analyses were stratified by sex and ethnicity. ACE2 activity was examined in Black and White men and women, and associations with endothelial dysfunction and oxidative stress markers were investigated in these sex and ethnic groups. A 2‐sided *P* value <0.05 was the criteria for statistical significance.

## RESULTS

Our population was primarily stratified by sex (Table 1). Men had higher clinic and ambulatory BP (all *P*<0.001) compared with women, and the prevalence of smoking was also higher among men than in women (*P*<0.001). Serum ACE2 activity was higher in men compared with women (*P*<0.001) (Figure 2A), and higher in Black men and women compared with their White counterparts (men: *P*<0.001; women: *P*=0.011) (results not shown in tables). We additionally performed analyses of covariance adjusting for ethnicity, smoking status, and BP to assess whether higher ACE2 activity would persist in men, and the results were consistent (*P*<0.001) (results not shown in tables). Men had higher levels of ADAMTS13 (*P*=0.015) (Figure 2B) and symmetric dimethylarginine (*P*<0.001) (Figure 2C) when compared with women. We found no differences in other markers of endothelial dysfunction (vWF [*P*=0.18] [Figure 2D] and ADMA [*P*=0.96] [Figure 2E]) between the 2 groups. Women had higher ROS (*P*<0.001) (Figure 2F) and malondialdehyde (*P*=0.035) (Figure 2G), but lower GGT (*P*<0.001) (Figure 2H), GPx (*P*<0.001) (Figure 2I), and total antioxidant status (*P*<0.001) (Figure 2J) compared with men. Other antioxidant capacity markers (SOD [*P*=0.26] [Figure 2K], GR [*P*=0.24] [Figure 2L], and total glutathione [*P*=0.06] [Figure 2M]) did not differ between men and women.

**Table 1:** Characteristics of Men and Women

	Men (N=557)	Women (N=577)	*P* value
Age, y	24.6±3.0	24.6±3.2	0.96
Ethnicity, Black, n (%)	280 (50.3)	285 (49.4)	0.77
ACE2 activity, undetectable, n (%)	31 (5.6)	252 (43.7)	<0.001*
Socioeconomic status score	20.6±6.2	20.9±6.1	0.47
Socioeconomic status classes, n (%)			0.23
Low	232 (41.7)	212 (36.7)	
Middle	151 (27.2)	172 (29.8)	
High	173 (31.1)	193 (33.4)	
Body composition			
Height, cm	174.6±7.9	162.9±7.1	<0.001*
Weight, kg	74.9±17.3	68.1±15.5	<0.001*
Body mass index, kg/m^2^	24.5±4.9	25.7±5.7	<0.001*
Waist circumference, cm	82.7±11.7	77.8±11.7	<0.001*
Blood pressure measurements			
Clinic systolic blood pressure, mm Hg	123±10.5	112±9.7	<0.001*
Clinic diastolic blood pressure, mm Hg	80±8.1	77±7.3	<0.001*
Clinic mean arterial pressure, mm Hg	97±8.2	91±7.6	<0.001*
24‐h mean arterial pressure, mm Hg	90±6.1	86±6.3	<0.001*
24‐h systolic blood pressure, mm Hg	122±8.0	113±8.5	<0.001*
24‐h diastolic blood pressure, mm Hg	70±6.1	68±5.6	<0.001*
24‐h heart rate, bpm	70±9.0	79±10.0	<0.001*
Basic biochemical variables			
Glucose, mmol/L	5.2 (4.9–5.4)	5.0 (4.7–5.2)	<0.001*
Glycated hemoglobin, %	5.3±0.3	5.4±0.3	0.006*
Total cholesterol, mmol/L	3.6±1.3	3.9±1.1	<0.001*
Low‐density lipoprotein, mmol/L	2.2 (1.6–3.0)	2.4 (1.9–3.0)	0.005*
High‐density lipoprotein, mmol/L	1.0±0.4	1.3±0.4	<0.001*
Triglycerides, mmol/L	0.7 (0.5–1.1)	0.7 (0.5–0.9)	0.021*
C‐reactive protein, mg/L	0.6 (0.2–1.5)	1.3 (0.4–4.0)	<0.001*
Cotinine, ng/mL	1.0 (1.0–129.5)	1.00 (1.00–1.00)	<0.001*
eGFR, mL/min per 1.73 m^2^	127.7±24.4	117.7±20.9	<0.001*
uACR, mg/mmol	0.4 (0.3–0.7)	0.5 (0.3–0.9)	<0.001*
Lifestyle factors			
AEE, kcal/kg per d	5.0±2.4	6.7±3.0	<0.001*
Current smoker, n (%)	191 (34.4)	74 (12.8)	<0.001*
Current alcohol use, n (%)	328 (59.3)	299 (52.2)	0.016*
Hormonal contraceptives			
Contraceptive pill, n (%)	…	151 (26.2)	…
Contraceptive injection, n (%)	…	95 (16.5)	…
Contraceptive implant, n (%)	…	31 (5.4)	…
Intrauterine device, n (%)	…	10 (1.7)	…
Hormone replacement therapy, n (%)	…	6 (1.0)	…

Values are expressed as mean±SD, median (25th–75th percentiles), or n (%), number of participants and proportions. *P* values are from 2‐sample *t* tests for normally distributed variables, Mann‐Whitney *U* tests for variables that were not normally distributed, or χ^2^ tests for categorical variables. Socioeconomic status was determined using an adapted Kuppuswamy scale for South Africa,^31^ including skill level, education, and household income, and categorized as low, middle, or high. Detailed methods are described elsewhere.^30^, ^32^ AEE was measured using an ActiHeart device worn for up to 7 days and was standardized for each participant's body weight for further analysis.

ACE2 indicates angiotensin‐converting enzyme 2; AEE, activity energy expenditure; eGFR, estimated glomerular filtration rate; and uACR, urinary albumin‐creatinine ratio.

* *P*<0.05.

**Figure 2:**
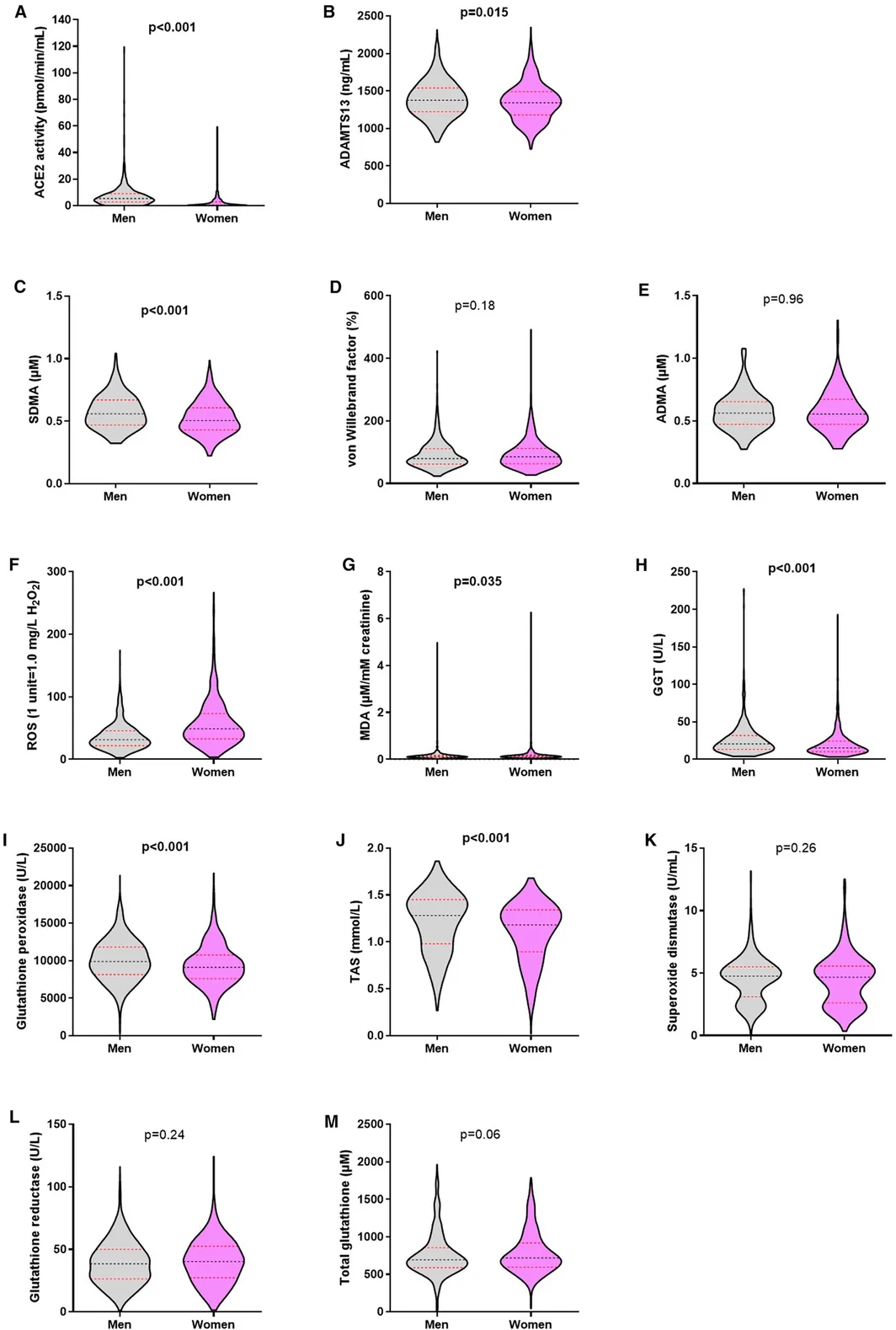
Comparison of ACE2 activity, endothelial dysfunction, and oxidative stress‐related markers between men and women. Violin plots illustrate the distribution of (**A**) ACE2 activity, (**B**) ADAMTS13, (**C**) SDMA, (**D**) von Willebrand factor, (**E**) ADMA, (**F**) ROS, (**G**) MDA, (**H**) GGT, (**I**) glutathione peroxidase, (**J**) TAS, (**K**) superoxide dismutase, (**L**) glutathione reductase; and (**M**) total glutathione in men (gray) and women (purple). Dotted lines in a contrasting color (red) indicate the 25th to 75th percentiles, whereas the median is indicated by the central dotted lines (black). *P* values are from 2‐sample *t* tests and Mann‐Whitney *U* tests. A total of 1134 participants were included in the analyses (men: N=557; women: N=577). ACE2 indicates angiotensin‐converting enzyme 2 activity; ADAMTS13, a disintegrin‐like metalloproteinase with thrombospondin motif type 1 member 13; ADMA, asymmetric dimethylarginine; GGT, gamma glutamyl transferase; MDA, malondialdehyde; ROS, reactive oxygen species; SDMA, symmetric dimethylarginine; and TAS, total antioxidant status.

Multivariable‐adjusted Tobit regression analysis with ACE2 activity as the dependent variable (Table 2), revealed a positive association with vWF (β=0.001, *P*=0.006) and ADAMTS13 (β=0.0002, *P*=0.019) in men. We observed a negative association of ACE2 activity with ADMA (β=−0.481, *P*=0.043) and SOD (β=−0.044, *P*=0.004) in women only. In both men and women, ACE2 activity was positively associated with GGT (men: β=0.007, *P*<0.001; women: β=0.008, *P*<0.001) and GR (men: β=0.004, *P*=0.004; women: β=0.004, *P*=0.039).

**Table 2:** Tobit Regression Analysis of the Associations of Natural Logarithm of ACE2 Activity With Markers of Endothelial Dysfunction and Oxidative Stress for Men and Women

	Men (N=557)		Women (N=577)	
	ACE2 activity (pmol/mL per min)			
	β (95% CI)	*P* value	β (95% CI)	*P* value
von Willebrand factor, %	0.001 (0.0004 to 0.003)	0.006*	0.001 (−0.0001 to 0.002)	0.08
ADAMTS13, ng/mL	0.0002 (0.000 to 0.0004)	0.019*	0.0001 (−0.0001 to 0.0003)	0.39
Asymmetric dimethylarginine, μM	−0.321 (−0.706 to 0.065)	0.10	−0.481 (−0.946 to −0.016)	0.043*
Symmetric dimethylarginine, μM	−0.194 (−0.621 to 0.233)	0.37	−0.347 (−0.909 to 0.216)	0.23
ROS (1 unit=1.0 mg/L H_2_O_2_)	0.001 (−0.001 to 0.003)	0.53	0.001 (−0.001 to 0.003)	0.33
Malondialdehyde, μM/mM creatinine	0.022 (−0.091 to 0.135)	0.70	−0.024 (−0.120 to 0.072)	0.62
γ Glutamyl transferase, U/L	0.007 (0.005 to 0.009)	<0.001*	0.008 (0.005 to 0.011)	<0.001*
Total antioxidant status, mmol/L	0.100 (−0.035 to 0.234)	0.15	0.150 (−0.052 to 0.353)	0.15
Superoxide dismutase, U/mL	−0.011 (−0.035 to 0.013)	0.37	−0.044 (−0.075 to −0.014)	0.004*
Glutathione peroxidase, U/L	0.000 (−0.000 to 0.000)	0.99	0.000 (−0.000 to 0.000)	0.10
Glutathione reductase, U/L	0.004 (0.001 to 0.006)	0.004*	0.004 (0.0002 to 0.007)	0.039*
Total glutathione, μM	0.000 (−0.0001 to 0.0002)	0.55	0.0002 (−0.000 to 0.0004)	0.08

Data are expressed as β coefficients and 95% CIs. Covariates included for each model were age, ethnicity, socioeconomic status class, body mass index, 24‐hour systolic blood pressure, glucose, low‐density lipoprotein, smoking status, and alcohol use. Hormonal contraceptive use was additionally adjusted for in women. *P* values are from multivariable‐adjusted Tobit regression analysis. ACE2 indicates angiotensin‐converting enzyme 2; ADAMTS13, a disintegrin‐like metalloproteinase with thrombospondin motif type 1 member 13; and ROS, reactive oxygen species.

* *P*<0.05.

When participants were stratified by sex and ethnicity (Table S2), we found a positive association of ACE2 activity with vWF in Black men (β=0.002, *P*=0.018) and women (β=0.001, *P*=0.038) and ADAMTS13 (β=0.0002, *P*=0.036) in Black men only. In White men, ACE2 activity associated positively with GR (β=0.005, *P*=0.015). ACE2 activity was inversely associated with SOD in Black (β=−0.052, *P*=0.023) and White (β=−0.042, *P*=0.042) women, but positively with total glutathione in Black women (β=0.0004, *P*=0.004) but also in White men (β=0.0004, *P*=0.002). In both Black and White men and women, ACE2 activity was positively associated with GGT (both *P*≤0.004).

Results in the total group (Table S3), align with the direction of associations observed in the sex groups, although we found no interactions between sex and markers of endothelial dysfunction and oxidative stress(Table S4), suggesting a consistent trend across subgroup analysis. ACE2 activity was independently associated with markers of endothelial dysfunction (vWF: β=0.001, *P*=0.001; ADAMTS13: β=0.0001, *P*=0.042; ADMA: β=−0.375, *P*=0.015) and oxidative stress (GGT: β=0.008, *P*<0.001) as well as GR (β=0.0001, *P*=0.001). Furthermore, ACE2 activity was negatively associated with SOD (β=−0.026, *P*=0.006) in the total group.

### Sensitivity Analysis

We performed a sensitivity analysis to assess the role of inflammation in the associations of ACE2 activity with markers of endothelial dysfunction and oxidative stress. In multivariable‐adjusted Tobit regression models additionally adjusting for C‐reactive protein, all observed associations in men and women remained significant whether C‐reactive protein was included or not (all *P*≤0.045) (Table S5).

## DISCUSSION

In this young healthy cohort, we found higher circulating ACE2 activity in men than in women, which was independent of BP and smoking status. ACE2 activity was associated with markers of endothelial dysfunction (vWF, ADAMTS13, ADMA) and an antioxidant enzyme (SOD). These associations varied between men and women.

Previous studies in individuals with CKD have reported higher circulating ACE2 activity in men than women,^28^, ^29^ which is in line with our findings, although our study population included younger and apparently healthy individuals. This suggests that young men in this study may have high CVD risk than women as reflected by higher ACE2 activity, which is associated with increased cardiovascular risk. The *ACE2* gene is located on the X chromosome. Typically, 1 of the female X chromosomes undergoes random inactivation to ensure equal gene expression between sexes, a process known as X‐inactivation.^43^ However, ACE2 is located within a region known to harbor genes that may escape X‐inactivation,^44^, ^45^ which may contribute to sex differences in ACE2 expression or activity.^46^ Although this has been proposed as a mechanism that could influence sex differences in ACE2 expression or activity, this phenomenon does not account for higher ACE2 activity observed in men in previous studies and our current observation. This observation could be partly explained by the well‐known high cardiovascular risk in men including elevated BP,^47^, ^48^ although the higher ACE2 activity observed in men in our study remained significant even after adjusting for BP. This indicates that other cardiometabolic and environmental exposures may contribute to the observed sex differences in ACE2 activity.

Distinct profiles of oxidative stress‐related markers were also evident between sexes. We observed higher levels of ROS and malondialdehyde in women, but lower GGT compared with men. Furthermore, total antioxidant status and GPx activity were lower in women than in men. A study by Ide et al^49^ showed that other markers of oxidative stress (plasma thiobarbituric acid‐reactive substances and urinary 8‐isoprostaglandin) were higher, whereas GPx activity was lower in young men than in premenopausal women (aged 20–39 years). However, that study was conducted in a smaller sample size (men: n=52; women: n=51).^49^ Women in our study may exhibit an increased generation of ROS and lipid peroxidation, which could be linked to the use of hormonal contraceptives.^41^, ^42^ Our results also indicate that women in our study may have an inadequate antioxidant defense capacity as reflected by lower GPx activity.

We observed similar levels of vWF between both sexes in our study; however, ACE2 activity was positively associated with vWF only in men. vWF plays an important role in platelet adhesion and aggregation under shear stress,^50^ with elevated vWF levels found in individuals with CVD risk factors or established CVD compared with healthy individuals.^20^ Higher levels of vWF are also shown to predict adverse cardiac events in patients with established CVD.^51^ The release of vWF is triggered by inflammatory cytokines stimuli,^52^ and inflammation is known to impair endothelial function, contributing to the early development of atherosclerosis.^53^, ^54^ In our study, ACE2 activity may be increased in response to vWF release and reflect a vascular environment prone to endothelial dysfunction, which in turn may increase the risk of thrombosis. Men also had higher ADAMTS13 levels compared with women, consistent with previous reports.^55^, ^56^, ^57^ Furthermore, higher levels of ADAMTS13 were associated with increased ACE2 activity in men. Under shear stress conditions, ADAMTS13 cleaves vWF to prevent excessive platelet adhesion and thrombosis.^58^ Our data suggest a potential trend of susceptibility to endothelial damage in men. Furthermore, ADAMTS13 levels may be elevated to potentially reduce the risk of thrombosis associated with vWF release and increased ACE2 activity.

In women, ADMA was negatively associated with ACE2 activity. Although ADMA levels did not differ between women and men, ACE2 activity was lower in women compared with men, indicating that the observed relationship may not be attributed to ADMA levels. Endothelial dysfunction is characterized by reduced NO bioavailability, which may result from decreased synthesis or increased inactivation.^59^ This potent endogenous vasodilator (NO), is synthesized from the NO synthase substrate, L‐arginine,^60^ with elevated levels of ADMA known to inhibit NO synthase activity, and associated with reduced NO bioavailability.^61^ The negative association observed between ADMA and ACE2 activity may potentially reflect shared underlying physiological processes associated with NO bioavailability and regulation of ACE2 activity. However, this interpretation remains speculative, because ADMA concentrations were measured in urine, whereas ACE2 activity was determined in serum, and the observed association is unlikely to represent a direct relationship between the 2 biomarkers.

SOD was negatively associated with ACE2 activity in women. SOD is known to be the first line of defense against the effects of superoxide anion,^62^ and the upregulation of SOD may be needed to increase the dismutation of superoxide anion to H_2_O_2_, to aid in the prevention of molecular oxidative damage.^62^ However, SOD activity was similar between men and women in our study. As oxidative stress increases, ACE2 activity may also increase, and SOD activity may be downregulated, suggesting that this antioxidant alone may not be sufficient to counteract the effects of oxidative stress alongside increased ACE2 activity. This highlights the importance of maintaining antioxidant defense systems to reduce oxidative stress and higher ACE2 activity.

The role of circulating ACE2 activity in oxidative stress among young healthy populations remains underresearched. To the best of our knowledge, this is the first study with a large sample size (N=1134) reporting associations between circulating ACE2 activity and markers of oxidative stress in a young healthy population. In both men and women, ACE2 activity was positively associated with GGT and GR. Although GGT is a widely recognized clinical biomarker of liver dysfunction and excessive alcohol consumption,^63^ evidence has highlighted its role as a circulatory biomarker for redox status.^64^ GGT enzymatic activity is involved in the metabolism of glutathione and contributes to the regulation of redox state in both intracellular and extracellular environments.^65^ In contrast, GGT also generates ROS during the process of glutathione metabolism, which supports the notion that this marker acts as both an antioxidant and a prooxidant.^66^ GR complements GGT by preserving the intracellular glutathione, to maintain the redox balance.^67^ Given the complex role of GGT, the consistent association of ACE2 activity with GGT and GR in both sexes indicate a complex interaction between ACE2 activity, oxidative stress, and redox regulation, although different mechanisms may be at play in men and women separately.

Membrane‐bound ACE2 acts locally within tissues to degrade angiotensin II and generate a bioactive angiotensin heptapeptide (angiotensin [1–7]), which has vasodilatory and anti‐inflammatory properties.^16^, ^17^ Several studies have shown that membrane‐bound ACE2 undergoes shedding, resulting in the soluble form released in the circulation.^20^, ^21^ Increased shedding occurs in conditions associated with inflammation.^68^, ^69^, ^70^ Therefore, elevated circulating ACE2 may not indicate enhanced local angiotensin II metabolism but may reflect reduced tissue ACE2 expression. Ramchand et al^24^ have shown that reduced tissue ACE2 expression is associated with elevated circulating ACE2 activity, which may reflect increased shedding of ACE2 in the circulation. Although bioengineered soluble ACE2 proteins have demonstrated therapeutic potential by binding and clearing circulating angiotensin II, generating a bioactive angiotensin heptapeptide (angiotensin [1–7]), and acting as a decoy receptor for SARS‐CoV‐2, these effects occur under experimental conditions.^71^ In contrast, our study measured endogenous circulating ACE2 activity in an apparently healthy population, which reflects physiological or pathological processes occurring in the body rather than treatment effects. This distinction is important when interpreting our findings. Increased shedding of ACE2 may elevate circulating ACE2 activity, and tissue ACE2 expression may be reduced, thereby reducing its local protective effects. However, the role of circulating ACE2 as an early biomarker of CVD risk in a young population warrants further research.

This study shows sex differences in circulating ACE2 activity among young adults and offers novel insights into sex‐specific associations between ACE2 activity and markers of endothelial dysfunction and oxidative stress as illustrated in Figure 3. A key strength of this study is its relatively large cohort of young, apparently healthy men and women, making it one of the first major studies to comprehensively examine sex differences in ACE2 activity before the onset of CVD. By evaluating associations with various markers of endothelial dysfunction and oxidative stress, this study contributes to a better understanding of circulating ACE2 activity as a potential early biomarker of CVD risk and its relationship with endothelial dysfunction and oxidative stress markers, which are known to contribute to CVD development. Importantly, sex‐specific patterns in serum ACE2 activity and its associations may have clinical relevance, because these findings highlight the need for sex‐specific approaches when evaluating early biomarkers or therapeutic strategies targeting RAAS‐related pathways. Our study also has several limitations. The observed associations between ACE2 activity and markers of endothelial dysfunction and oxidative stress were generally modest; therefore, the findings should be interpreted with appropriate caution. The cross‐sectional design limits causal inference, and our results do not establish circulating ACE2 as a primary mechanism underlying endothelial dysfunction. Although circulating ACE2 activity has been linked to inflammatory processes, this study design cannot determine with certainty whether elevated ACE2 activity contributes to vascular alterations, including inflammation, endothelial dysfunction, and oxidative stress, or whether these vascular changes promote increased ACE2 shedding in circulation. Longitudinal and mechanistic studies are required to clarify causal relationships linking ACE2 activity with endothelial dysfunction and oxidative stress. In addition, although angiotensin‐converting enzyme is an integral component of the RAAS, the angiotensin‐converting enzyme‐to‐ACE2 activity ratio was not assessed in this study, because only angiotensin‐converting enzyme concentration as opposed to activity data were available and thus not included. Enzyme concentration and activity are not directly comparable measures, therefore, this limited a comprehensive evaluation of possible RAAS dysregulation. Finally, certain biomarkers, such as malondialdehyde, ADMA, and symmetric dimethylarginine, were measured in urine, which may not precisely reflect their relationship with circulating ACE2 activity.

**Figure 3:**
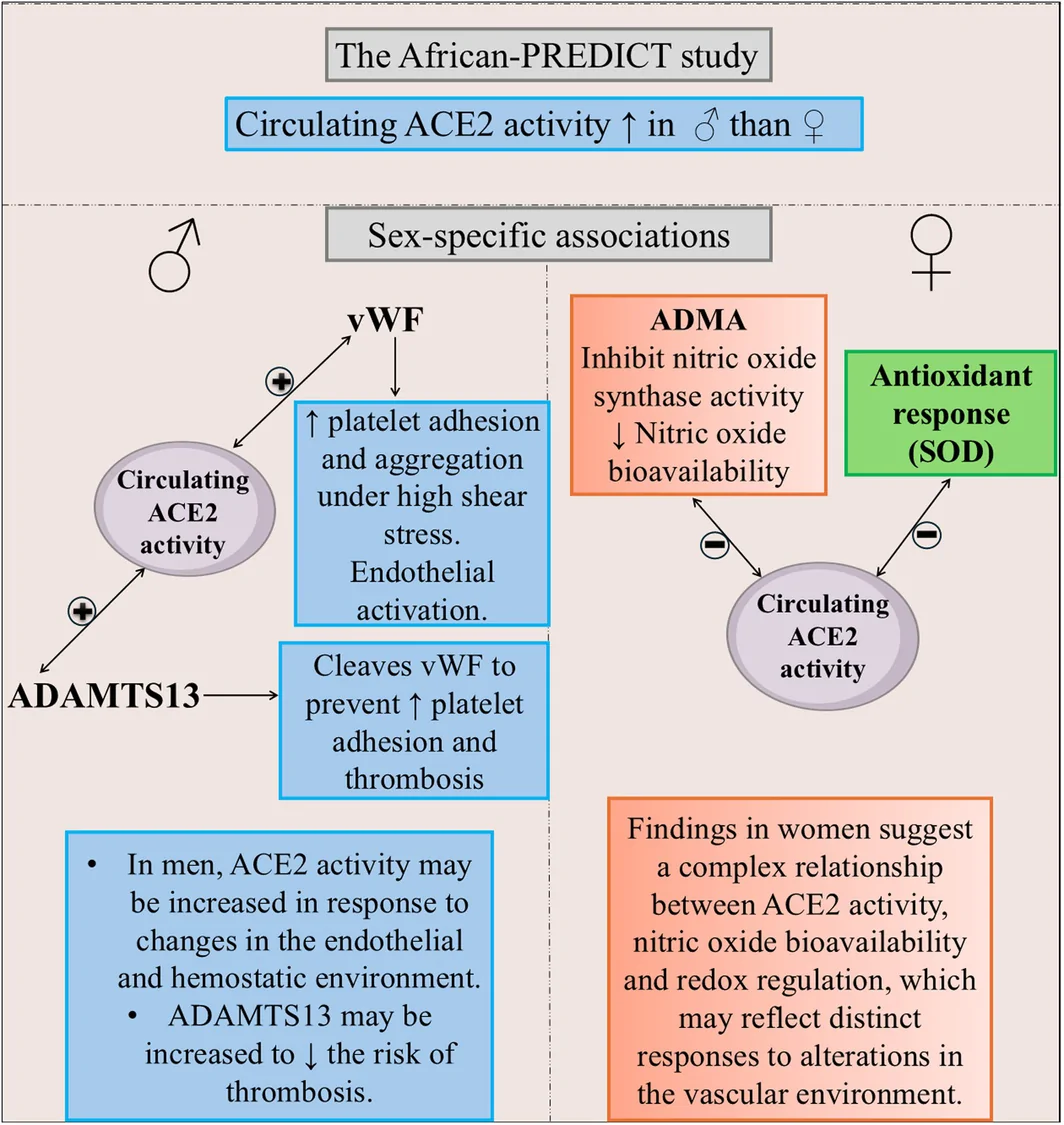
Schematic illustration of the relationship between circulating ACE2 activity, endothelial dysfunction, and oxidative stress. The schema summarizes study findings and proposes biological mechanisms of the observed associations of circulating ACE2 activity with markers of endothelial dysfunction and oxidative stress. Positive and negative associations observed in the study are shown as ↔, + and −, respectively, highlighting sex‐specific relationships (men ♂, women ♀). ↑ indicates higher/increased, whereas ↓ indicates reduced/decreased. The ↔ symbol mean association. This was used for the purpose of differentiating the association arrows with the other arrow. ACE2 indicates angiotensin‐converting enzyme 2; ADAMTS13, a disintegrin‐like metalloproteinase with thrombospondin motif type 1 member 13; ADMA, asymmetric dimethylarginine; African‐PREDICT, African Prospective Study on Early Detection and Identification of Cardiovascular Disease and Hypertension; SOD, superoxide dismutase; and vWF, von Willebrand factor.

## CONCLUSIONS

In young apparently healthy adults, serum ACE2 activity was higher in men than in women, independent of BP and smoking status, and associated with endothelial dysfunction and oxidative stress markers in men and women, respectively. The positive association of ACE2 activity with vWF and ADAMTS13 in men suggests that higher ACE2 activity may be linked to endothelial and hemostatic processes, whereas the negative association with ADMA and SOD in women suggest a complex relationship between ACE2 activity, NO bioavailability, and redox regulation. These findings in an apparently healthy population provide a unique pathophysiological role of ACE2 activity as potential early biomarker of CVD risk.

## Sources of Funding

This work was supported by the South African Medical Research Council (SAMRC) with funds from National Treasury under its Economic Competitiveness and Support Package; the South African Research Chairs Initiative (SARChI) of the Department of Science and Technology and National Research Foundation (NRF) of South Africa [GUN 86895]; SAMRC with funds received from the South African National Department of Health, GlaxoSmithKline R&D (Africa Non‐Communicable Disease Open Lab grant), the UK Medical Research Council and with funds from the UK Government's Newton Fund; as well as corporate social investment grants from Pfizer (South Africa), Boehringer‐Ingelheim (South Africa), Novartis (South Africa), the Medi Clinic Hospital Group (South Africa) and in‐kind contributions of Roche Diagnostics (South Africa). Funding for the ACE2 activity assays was supported by a National Health and Medical Research Council Program Grant [APP1055214 to L.B]. This work is based on the research supported in part by the NRF of South Africa. Any opinion, findings, and conclusions or recommendations expressed in this material are those of the authors, and therefore, the NRF does not accept any liability in this regard.

## Disclosures

None.

## Supporting information

Tables S1–S5

STROBE Checklist
