# Supplementary material for: Associations of Angiotensin‐Converting Enzyme 2 Activity With Markers of Endothelial Dysfunction and Oxidative Stress in Young Healthy Adults: The African‐PREDICT Study
Source: J Am Heart Assoc. 2026 Oct 16;15(20):e047345. doi: 10.1161/JAHA.125.047345 (PMC13769619; doi:10.1161/JAHA.125.047345)
Supplement: Supplementary file 2 [file JAH3-15-e047345-s002.pdf]

SUPPLEMENTAL MATERIAL

Table S1: Number of participants for each variable with missing values.

	**Number of participants with missing values**
**Endothelial dysfunction**	
von Willebrand factor (%)	n=61
ADAMTS13 (ng/mL)	n=8
Asymmetric dimethylarginine (μM)	n=585
Symmetric dimethylarginine (μM)	n=586
**Oxidative stress-related markers**	
Reactive oxygen species (1 unit = 1.0 mg/L H_2O_2)	n=1
Malondialdehyde (μM/mM creatinine)	n=10
Gamma glutamyl transferase (U/L)	n=3
Total antioxidant status (mmol/L)	n=1
Superoxide dismutase (U/mL)	n=4
Glutathione peroxidase (U/L)	n=2
Glutathione reductase (U/L)	n=2
Total glutathione (μM)	n=4

*ADAMTS13, a disintegrin-like metalloproteinase with thrombospondin motif type 1 member 13.

Table S2: Tobit regression analysis of the associations of natural logarithm of ACE2 activity with markers of endothelial dysfunction and oxidative stress for Black and White men and women

	Men (N=557)				Women (N=577)			
	Black (n=280)		White (n=277)		Black (n=285)		White (n=292)	
	ACE2 activity (pmol/min/mL)							
**Endothelial dysfunction**	***β (95% CI)***	***p***	***β (95% CI)***	***p***	***β (95% CI)***	***p***	***β (95% CI)***	***p***
vWF (%)	0.002 (0.0003; 0.003)	**0.018**	0.001 (-0.0002; 0.003)	0.09	0.001 (0.0001; 0.003)	**0.038**	-0.0003 (-0.002; 0.002)	0.81
ADAMTS13 (ng/mL)	0.0002 (0.000; 0.001)	**0.036**	0.0002 (-0.0001; 0.001)	0.11	0.0002 (-0.0002; 0.001)	0.32	0.000 (-0.0003; 0.0004)	0.77
ADMA (µM)	-0.083 (-0.586; 0.420)	0.75	-0.523 (-1.107; 0.060)	0.08	-0.577 (-1.163; 0.009)	0.053	-0.158 (-0.940; 0.624)	0.69
SDMA (µM)	-0.300 (-0.892; 0.292)	0.32	-0.120 (-0.720; 0.481)	0.70	-0.142 (-0.831; 0.547)	0.69	-0.583 (-1.549; 0.384)	0.24
**Oxidative stress**								
ROS	0.001 (-0.001; 0.003)	0.34	-0.0002 (-0.004; 0.003)	0.90	0.0004 (-0.002; 0.003)	0.69	0.002 (-0.001; 0.004)	0.25
MDA	-0.003 (-0.216; 0.209)	0.98	0.042 (-0.094; 0.179)	0.54	0.075 (-0.148; 0.298)	0.51	-0.071 (-0.174; 0.032)	0.18
GGT (U/L)	0.005 (0.002; 0.007)	**<0.0001**	0.011 (0.007; 0.014)	**<0.001**	0.007 (0.004; 0.011)	**<0.001**	0.013 (0.006; 0.020)	**0.0004**
TAS (mmol/L)	0.087 (-0.091; 0.264)	0.34	0.108 (-0.092; 0.308)	0.29	0.124 (-0.192; 0.440)	0.44	0.210 (-0.056; 0.476)	0.12
SOD (U/mL)	-0.009 (-0.040; 0.023)	0.59	-0.018 (-0.053; 0.017)	0.31	-0.052 (-0.096; -0.007)	**0.023**	-0.042 (-0.083; -0.002)	**0.042**
GPx (U/L)	0.000 (-0.000; 0.000)	0.27	-0.000 (-0.000; 0.000)	0.23	0.000 (-0.000; 0.000)	0.43	0.000 (-0.000; 0.0001)	0.12
GR (U/L)	0.003 (-0.001; 0.006)	0.13	0.005 (0.001; 0.008)	**0.015**	0.003 (-0.002; 0.008)	0.17	0.004 (-0.001; 0.009)	0.08
tGSH (µM)	-0.0001 (-0.0003; 0.000)	0.16	0.0004 (0.0002; 0.001)	**0.002**	0.0004 (0.0001; 0.001)	**0.004**	-0.0003 (-0.001; 0.0001)	0.20

*Data expressed as β-coefficient and 95% confidence interval. Covariates included for each model were age, socioeconomic status class, body mass index, 24-hour systolic blood pressure, glucose, low density lipoprotein, smoking status, and alcohol use. Hormonal contraceptive use was additionally adjusted for in women. p-values are from multivariable-adjusted Tobit regression analysis. Bold p-value indicates $p<0.05$.

†ACE2, angiotensin converting enzyme 2; vWF, von Willebrand factor; ADAMTS13, a disintegrin-like metalloproteinase with thrombospondin motif type 1 member 13; ADMA, asymmetric dimethylarginine; SDMA, symmetric dimethylarginine; ROS, reactive oxygen species (1 unit = 1.0 mg/L H_2O_2); MDA, malondialdehyde (μM/mM creatinine); GGT; gamma glutamyl transferase; TAS, total antioxidant status; SOD, superoxide dismutase; GPx, glutathione peroxidase; GR, glutathione reductase; tGSH, total glutathione.

Table S3: Tobit regression analysis of the associations of natural logarithm of ACE2 activity with markers of endothelial dysfunction and oxidative stress for the total group

	**Total group (N=1134)** **ACE2 activity (pmol/min/mL)**	
	β (95% CI)	***p-value***
von Willebrand factor (%)	0.001 (0.001; 0.002)	**0.001**
ADAMTS13 (ng/mL)	0.0001 (0.000; 0.0003)	**0.042**
Asymmetric dimethylarginine (µM)	-0.375 (-0.677; -0.073)	**0.015**
Symmetric dimethylarginine (µM)	-0.196 (-0.543; 0.152)	0.27
ROS (1 unit = 1.0 mg/L H_2O_2)	0.001 (-0.001; 0.002)	0.25
Malondialdehyde (µM/mM creatinine)	-0.010 (-0.082; 0.062)	0.79
Gamma glutamyl transferase (U/L)	0.008 (0.006; 0.009)	**<0.001**
Total antioxidant status (mmol/L)	0.100 (-0.014; 0.213)	0.09
Superoxide dismutase (U/mL)	-0.026 (-0.045; -0.008)	**0.006**
Glutathione peroxidase(U/L)	0.000 (-0.000; 0.000)	0.15
Glutathione reductase (U/L)	0.003 (0.001; 0.005)	**0.001**
Total glutathione (µM)	0.0001 (-0.000; 0.0002)	0.12

*Data expressed as β-coefficient and 95% confidence interval. Covariates included for each model were age, sex, ethnicity, socioeconomic status class, body mass index, 24-hour systolic blood pressure, glucose, low density lipoprotein, smoking status, and alcohol use. p-values are from multivariable-adjusted Tobit regression analysis. Bold p-value indicates $p<0.05$.

†ACE2, angiotensin converting enzyme 2; ADAMTS13, a disintegrin-like metalloproteinase with thrombospondin motif type 1 member 13; ROS, reactive oxygen species.

Table S4: Tobit regression analysis of the associations of natural logarithm of ACE2 activity with markers of endothelial dysfunction and oxidative stress for the total group

**Total group (N=1134)**		
**ACE2 activity (pmol/min/mL)**		
	β (95% CI)	***p-value***
Sex x von Willebrand factor (%)	0.001 (-0.001; 0.002)	0.38
Sex x ADAMTS13 (ng/mL)	0.000 (-0.0002; 0.0003)	0.76
Sex x asymmetric dimethylarginine (μM)	0.290 (-0.284; 0.863)	0.32
Sex x symmetric dimethylarginine (μM)	0.112 (-0.575; 0.798)	0.75
Sex x ROS (1 unit = 1.0 mg/L H_2O_2)	0.000 (-0.003; 0.003)	0.99
Sex x malondialdehyde (μM/mM creatinine)	0.051 (-0.098; 0.200)	0.50
Sex x gamma glutamyl transferase (U/L)	-0.001 (-0.004; 0.002)	0.49
Sex x total antioxidant status (mmol/L)	-0.050 (-0.281; 0.180)	0.67
Sex x superoxide dismutase (U/mL)	0.035 (-0.002; 0.071)	0.06
Sex x glutathione peroxidase(U/L)	0.000 (-0.000; 0.000)	0.23
Sex x glutathione reductase (U/L)	0.001 (-0.003; 0.005)	0.74
Sex x total glutathione (μM)	-0.0001 (-0.0003; 0.0002)	0.52

*Data expressed as β-coefficient and 95% confidence interval. Covariates included for each model were age, sex, ethnicity, socioeconomic status class, body mass index, 24-hour systolic blood pressure, glucose, low density lipoprotein, smoking status, and alcohol use. p-values are from multivariable-adjusted Tobit regression analysis.

†ACE2, angiotensin converting enzyme 2; ADAMTS13, a disintegrin-like metalloproteinase with thrombospondin motif type 1 member 13; ROS, reactive oxygen species.

Sensitivity analysis

Table S5: Tobit regression analysis of the associations of natural logarithm of ACE2 activity with markers of endothelial dysfunction and oxidative stress for men and women

	**Men (N=557)**		**Women (N=577)**	
	**ACE2 activity (pmol/min/mL)**			
	β (95% CI)	***p-value***	***β (95% CI)***	***p-value***
von Willebrand factor (%)	0.001 (0.0004; 0.003)	**0.006**	0.001 (-0.0001; 0.002)	0.08
ADAMTS13 (ng/mL)	0.0002 (0.000; 0.0004)	**0.019**	0.0001 (-0.0001; 0.0003)	0.39
Asymmetric dimethylarginine (μM)	-0.318 (-0.703; 0.066)	0.10	-0.467 (-0.923; -0.011)	**0.045**
Symmetric dimethylarginine (μM)	-0.184 (-0.609; 0.242)	0.40	-0.272 (-0.826; 0.283)	0.34
ROS (1 unit = 1.0 mg/L H_2O_2)	0.001 (-0.001; 0.003)	0.57	0.001 (-0.001; 0.002)	0.39
Malondialdehyde (μM/mM creatinine)	0.024 (-0.089; 0.137)	0.68	-0.022 (-0.118; 0.074)	0.66
Gamma glutamyl transferase (U/L)	0.007 (0.005; 0.009)	**<0.001**	0.008 (0.005; 0.011)	**<0.001**
Total antioxidant status (mmol/L)	0.100 (-0.035; 0.234)	0.14	0.149 (-0.053; 0.352)	0.15
Superoxide dismutase (U/mL)	-0.011 (-0.034; 0.013)	0.38	-0.044 (-0.074; -0.014)	**0.005**
Glutathione peroxidase(U/L)	0.000 (-0.000; 0.000)	0.90	0.000 (-0.000; 0.000)	0.09
Glutathione reductase (U/L)	0.003 (0.001; 0.006)	**0.006**	0.004 (0.0002; 0.007)	**0.040**
Total glutathione (μM)	0.000 (-0.0001; 0.0002)	0.54	0.0002 (-0.000; 0.0004)	0.08

*Data expressed as β-coefficient and 95% confidence interval. Covariates included for each model were age, ethnicity, socioeconomic status class, body mass index, 24-hour systolic blood pressure, glucose, low density lipoprotein, smoking

status, and alcohol use. Hormonal contraceptive use was additionally adjusted for in women. p-values are from multivariable-adjusted Tobit regression analysis additionally adjusted for C-reactive protein. Bold p-value indicates $p<0.05$.

†ACE2, angiotensin converting enzyme 2; ADAMTS13, a disintegrin-like metalloproteinase with thrombospondin motif type 1 member 13; ROS, reactive oxygen species.
